# Exchange of C-Terminal Variable Sequences within Morbillivirus Nucleocapsid Protein Are Tolerated: Development and Evaluation of Two Marker (DIVA) Vaccines (Sungri/96 DIVA, Nigeria/75/1 DIVA) against PPR

**DOI:** 10.3390/v13112320

**Published:** 2021-11-21

**Authors:** Muneeswaran Selvaraj, Mana Mahapatra, Satya Parida

**Affiliations:** 1 The Pirbright Institute, Ash Road, Pirbright, Woking GU24 ONF, Surrey, UK; dr.muneeswaran@gmail.com (M.S.); mana.mahapatra@pirbright.ac.uk (M.M.); 2 Food and Agriculture Organization of the United Nations (FAO), Viale delle Terme di Caracalla, 00153 Rome, Italy

**Keywords:** peste des petits ruminants (PPR) virus, differentiation of infected from vaccinated animals (DIVA), Sungri/96 DIVA vaccine, Nigeria/75/1 DIVA vaccine, recombinant vaccine, PPR DIVA vaccine safety and potency, DIVA test, nucleocapsid protein

## Abstract

Across Africa, the Middle East, and Asia, peste des petits ruminants virus (PPRV) places a huge disease burden on agriculture, affecting, in particular, small ruminant production. The recent PPR outbreaks in Northern Africa, the European part of Turkey, and Bulgaria represent a significant threat to mainland Europe, as a source of disease. Although two safe and efficacious live attenuated vaccines (Sungri/96 and Nigeria/75/1) are available for the control of PPR, current serological tests do not enable the differentiation between naturally infected and vaccinated animals (DIVA). The vaccinated animals develop a full range of immune responses to viral proteins and, therefore, cannot be distinguished serologically from those that have recovered from a natural infection. This poses a serious problem for the post-vaccinal sero-surveillance during the ongoing PPR eradication program. Furthermore, during the latter stages of any eradication program, vaccination is only possible if the vaccine used is fully DIVA compliant. Using reverse genetics, we have developed two live attenuated PPR DIVA vaccines (Sungri/96 DIVA and Nigeria/75/1 DIVA), in which the C-terminal variable region of the PPRV N-protein has been replaced with dolphin morbillivirus (DMV). As a proof of principle, both the DIVA vaccines were evaluated in goats in pilot studies for safety and efficacy, and all the animals were clinically protected against the intranasal virulent virus challenge, similar to the parent vaccines. Furthermore, it is possible to differentiate between infected animals and vaccinated animals using two newly developed ELISAs. Therefore, these DIVA vaccines and associated tests can facilitate the sero-monitoring process and speed up the implementation of global PPR eradication through vaccination.

## 1. Introduction

Peste des petits ruminants (PPR) is a highly contagious and economically important viral disease of small ruminants, including goats, sheep, and wild ungulates, caused by the PPR virus (PPRV). Cattle and buffalo can also be infected sub-clinically, but are unable to transmit the disease to other animals [1]. PPRV is a great burden on the developing world as the disease prevents the development of sustainable agriculture. The disease is emerging in new regions of the world and is already endemic in Asia, the Middle East, and throughout Africa, except for some countries in Southern Africa [2]. After recent outbreak reports from the European part of Turkey, North African countries, and Bulgaria, there is now a major risk of the introduction of PPR to the European mainland [1,3,4]. PPRV belongs to the genus Morbillivirus in the family Paramyxoviridae, and is a single stranded negative sense RNA virus whose genome is approximately 16 kb, organized into eight genes in the order 3’-N-P/C/V-M-F-HN-L-5’, which encodes six structural proteins, namely nucleoprotein (N); phosphoprotein (P); matrix protein (M); fusion protein (F); hemagglutinin protein (HN); large polymerase (L) protein; and two non-structural proteins, namely C- and V-, which are derived from the P-gene open reading frame (ORF) [5,6].

Following on from the successful eradication of rinderpest in 2011, PPR is currently the target of an international eradication campaign which aims to eradicate the disease by 2030 [7]. Currently, two live attenuated vaccine strains (Nigeria/75/1 and Sungri/96) are being regularly employed against this disease in endemic regions with a very good success rate [8,9] for providing protection, but it is not possible to differentiate between infected and vaccinated populations. The existing commercially available diagnostic ELISA kits for N- and H-proteins detect antibodies in vaccinated as well as naturally infected animals. Therefore, no tools currently exist that allow the serological differentiation between infected and vaccinated animals (DIVA), warranting the development of a DIVA vaccine with an associated DIVA test that can be a very useful tool for the eradication process, especially at the final stage.

Reverse genetics provides a means to manipulate RNA virus genomes through DNA copies (cDNA) to obtain a modified form of the virus. Using this technique, the H- and F- glycoprotein genes, together or in conjunction with the M-gene in the rinderpest vaccine virus (RPV), were replaced with similar genes from the PPRV resulting in the generation of recombinant marker vaccines for PPRV (rRPV–PPRFH or rRPV–PPRMFH) [10,11]. However, after the eradication of rinderpest, these vaccines cannot be used as they contain the rinderpest genome in their vaccine formulations. Subsequently, Muniraju and colleagues (2015) recovered a recombinant PPRV/Nigeria/75/1 vaccine virus in which the epitope in the H-protein, recognized by the monoclonal antibody (mAb) C77 (used in the diagnostic H-cELISA), was modified with the aim of developing a DIVA vaccine (negative marker) for PPRV. An in vivo assessment of this vaccine revealed it to be safe with a potency similar to that of the parental live attenuated PPRV/Nigeria/75/1 vaccine. Although the epitope change prevented the binding of C77 mAb in vitro, it was not sufficient to enable DIVA in vivo, possibly because of the presence of overlapping epitopes [12]. Therefore, the development of marker vaccines for PPRV using similar/other novel strategies is warranted.

Among the structural proteins, the N-protein is antigenically the most conserved among morbilliviruses and is highly immunogenic despite its internal location [13,14]. It has also been used as a target for virus detection in clinical specimens, since it is expressed (gradient expression) to a very high level in morbillivirus infected cells because of its location close to the promoter [15,16,17]. Following an infection, the dominant antibody responses are mainly against the surface glycoprotein, H-, and the nucleocapsid protein, N-, but only the antibodies against the H-protein can neutralize the virus [18,19]. The alignment of morbillivirus N-protein amino acid (aa) sequences has defined four regions (I–IV) with varying degrees of sequence homology, in the following order: the highly conserved region III (aa 145–420) is followed by region I (aa 1–122), which is subsequently followed by region II (aa 123–144) and, finally, the least conserved region IV (aa 421–525), at the C-terminus of the protein. Parida and colleagues rescued a chimeric rinderpest virus (rRPV–PPRVN) in which the RPV N-gene ORF was replaced with that of PPRV [20]. The rRPV-PPRVN exhibited efficient replication in cell culture and suggested the possibility of using this virus as a marker vaccine in the rinderpest eradication program. Furthermore, the authors developed a rinderpest specific ELISA using the recombinant protein containing the most variable part of the N-protein (IV region) to enable DIVA. Similarly, in Newcastle Disease Virus (NDV) deletion/replacement of a conserved B cell immunodominant epitope at the C-terminus of the N-protein did not have any adverse effect on the replication efficiency or immunogenicity of the virus [21]. Epitope mapping studies [22] showed that the epitopes recognized by anti-N mAbs specific to Measles virus were located mainly in regions IV (C-terminal) and II, the least conserved areas of the protein. Therefore, variable region (IV) of the N-protein can be used for serological screening of naturally infected and vaccinated animals, although it does not provide humoral immune protection.

In this study, we report the development of a reverse genetics system for the lineage IV vaccine strain, Sungri/96 for the first time. In addition, the most variable part of Sungri/96 and Nigeria/75/1 PPRV N-protein (region-IV aa 406–525) was replaced with that from a related morbillivirus, Dolphin morbillivirus (DMV). The recombinant viruses exhibited growth characteristics in cell culture similar to those of the parent viruses, and animals vaccinated with these chimeric viruses were protected from intranasal challenge with virulent PPRV. Furthermore, two indirect ELISAs were developed using recombinant proteins that distinguish between vaccinated and infected animals.

## 2. Materials and Methods

### 2.1. Cells and Viruses

Vero cells or Vero Dog SLAM tag (VDS) cells constitutively expressing the canine morbillivirus receptor SLAM were used to grow PPRV and DMV, and for transfection experiments. The cells were maintained in Dulbecco’s modified Eagle’s medium (DMEM) supplemented with 25 mM HEPES (pH 7.2), 10% (*v/v*) foetal calf serum (FCS, Gibco, Waltham, MA, USA), penicillin (100 Units/mL, Sigma, St. Louis, MO, USA) and streptomycin (100 μg/mL, Sigma, St. Louis, MO, USA).

Vero or VDS cells at ~70% confluency were infected with the vaccine virus or the rescued recombinant virus at an multiplicity of infection (MOI) of 0.1 and incubated at 37 °C/5% CO2. When the cytopathic effect (CPE) was almost complete, the virus was harvested by one cycle of freezing and thawing followed by centrifugation at 1280× *g* for 10 min to remove cell debris. The rescued recombinant viruses were grown and titrated on VDS cells as described above. Recombinant fowlpox virus was grown in chicken embryo fibroblasts as previously described [10].

### 2.2. Plasmids and Molecular Biology Techniques

All the DNA manipulations and cloning were carried out using standard protocols. The plasmid pPPRV containing the full length antigenome sequence of lineage II PPRV/Nigeria/75/1 vaccine strain (renamed as pNigeria/75/1) and three helper plasmids, pN, pP, pL have been described elsewhere [12]. Using a similar strategy, a plasmid containing the complete PPRV antigenome sequence (15,948 nt) of the lineage IV vaccine strain, Sungri/96 (GenBank accession number KJ867542.1) with the insertion of eGFP gene (pSungri/96-GFP) was designed and synthesized commercially (DNA2.0, Newark, CA, USA). The eGFP reporter gene was introduced to enable rapid evaluation of rescue events, as a separate transcriptional unit between the P- and M-gene with the authentic 5′ untranslated regions (UTRs) of the M-gene and the 3′ UTR of the P-gene. Unique restriction enzyme sites inserted by nucleotide substitutions into the untranslated regions of each gene were the same as in the pNigeria/75/1 plasmid. The synthesized plasmid, pSungri/96-GFP was sequenced in its entirety to ensure the sequence was identical to the vaccine strain sequence. After the successful recovery of a recombinant PPRV expressing the eGFP gene, the eGFP gene in the plasmid pSungri/96-GFP was removed using M*lu*I restriction sites that flanked the eGFP gene to obtain plasmid, pSungri/96. This pSungri/96 plasmid along with pNigeria/75/1 were used subsequently for making chimeric full-length genome constructs. Total RNA was extracted from infected cultures using Trizol (Invitrogen, Carlsbad, CA, USA) as described previously [10]. The real-time reverse transcription polymerase chain reaction (RT-qPCR) was performed following the method described by [23]. DNA sequencing was performed using an ABI sequencing kit.

### 2.3. Cloning of the PPR–DMV Chimeric N-Gene

In order to manipulate the N-gene, the restriction sites *AclI* (at the beginning of the N-gene) and *PacI* (immediately after the ORF of the N-gene) of the full-length plasmids were used. A chimeric N-gene containing the first part from the PPRV (~1.2 kb) and second part from the DMV (360 nt) was generated using a two-step overlap PCR. Tables S1–S3 lists all the primers used for the construction of the different plasmids containing chimeric N-genes. The outside PCR primer, PPR–Acl IF, at the beginning of the PPRV N-gene contained the *AclI* restriction site. Similarly, the outside PCR primer, DMV–Pac IR, at the end of DMV N-gene contained the *PacI* restriction site. The inside primers were designed on the N-gene of the PPRV or DMV in such a way that both the forward and reverse primers contained the identical sequence (15 nt upstream and 19 nt downstream) in the overlapping region. The part of the PPRV N-gene (~1.2 kb) was amplified using the primer set PPR–Acl IF (GCG CAA GAT CTA ACG TTA TGG CGA CTC TCC)/DMV-PPRR (ACC TAT TGC TCT ATT AGC TCT TTC GTC CCC AGC C). Similarly, the part of the DMV N-gene (~360 bp) was amplified using the primer set PPR–DMVF (GGC TGG GGA CGA AAG AGC TAA TAG AGC AAT AGG T)/DMV–Pac IR (CGG CCT TAA TTA AAC GCT GCT CAG AGT GGA TCC). These PCR products were gel purified and used as the templates for overlapping PCR using primer set PPR–Acl IF/DMV–Pac IR. The final PCR products were ligated into pT7Blue blunt end vector (Merck Millipore, Darmstadt, Germany) to produce the intermediate plasmids, pT7PPRSungri-DMVN120 and pT7PPRNigeria-DMVN120 and these plasmids were sequenced on both the strands to ensure there were no PCR-induced mutations.

### 2.4. Construction of Full-Length Chimeric Genome Plasmids

**Figure 1:**
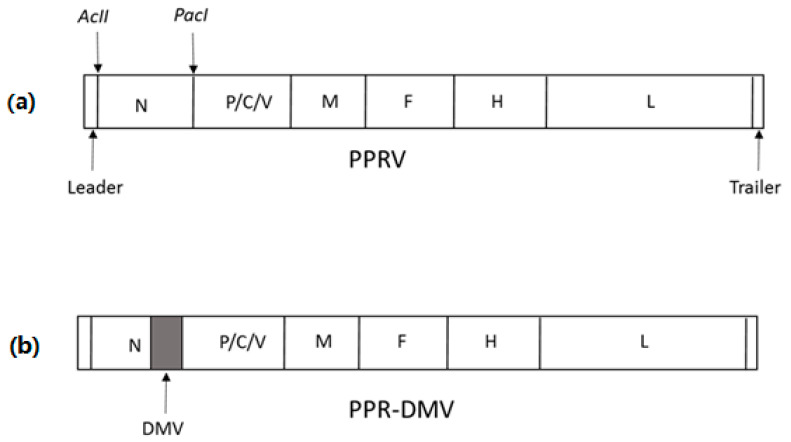
Schematic showing the genome of the PPRV (**A**) and PPR–DIVA (**B**) viruses (not to scale). The parental PPRV is shown in (**A**), indicating the unique restriction sites used for the construction of PPR–DIVA cDNA. The shaded box (**B**) represent the gene from DMV.

Two full-length chimeric genome plasmids were constructed in this study. The AclI/PacI digestion product of plasmids pT7PPRSungri-DMVN120 and T7pPPRNigeria-DMVN120 were used to replace the N-gene ORF of pSungri/96 and pNigeria/75/1 to make the full-length genome plasmids pSungri/96-DMV and pNigeria/75/1-DMV, respectively (Figure 1). Restriction enzyme analysis was carried out to ensure that the plasmids contained full-length copies of the viral genome followed by the sequencing of the entire chimeric N-gene to ensure that the sequence was correct.

### 2.5. Cloning of the C-Terminal Variable Region of the N-Gene of PPRV and DMV

The N-gene of PPRV and DMV encoding the last 120 amino acids at the C-terminus was amplified separately by PCR, using the primer sets PPRV-BamHIF1/PPRV-HindIIIR, and DMV-BamHIF1/DMV-HindIIIR (Supplementary Table S4), respectively. The PCR products were cloned into the pT7 blue blunt end vector generating the plasmids pT7-PPRVNv and pT7-DMVNv, respectively, and sequenced on both the strands to ensure that there were no PCR induced mutations. The BamHI/HindIII digestion product of the plasmid pT7-PPRVNv or pT7-DMVNv was ligated into the same sites of the bacterial expression vector pQE30Xa (Qiagen, Hilden, Germany) to produce the plasmids pQE30Xa–PPRVNv or pQE30Xa–DMVNv. This vector adds an N-terminal His-tag to the protein coding sequence.

### 2.6. Expression and Purification of His-Tagged Protein

To express the protein, the plasmids pQE30Xa–DMVNv and pQE30Xa–PPRVNv were transformed into *E. coli* strain M15 (Merck Millipore, Darmstadt, Germany) and grown in an LB medium containing ampicillin (50 mg/mL) and kanamycin (25 mg/mL). The protein expression was induced essentially, as described in the manufacturer’s protocol. The proteins (PPRVNv and DMVNv) were tested for their solubility and purified using Ni-NTA resin (Qiagen, Hilden, Germany). The protein samples were analyzed using 12% of SDS-PAGE. The concentration of the protein was determined using a protein assay kit (Bio-Rad, Hercules, CA, USA).

### 2.7. Transfection and Rescue of Recombinant PPRV from cDNA

Transfection experiments were carried out following the method of Muniraju and colleagues (2015). Briefly, Vero or VDS cells (~70% confluent) grown in 6-well plates were infected with a recombinant fowlpox virus that expressed the T7-RNA polymerase at an MOI of 0.2 for 1 h. The cells were washed and transfected with an appropriate full-length PPRV plasmid and helper plasmids, pN, pP, and pL, using the TransFastTM transfection reagent (Promega, Madison, WI, USA) at a ratio of 6:1 (*wt/wt*) in a total volume of 0.75 mL of OPTI-MEM I reduced serum medium/well (Gibco, Waltham, MA, USA). The cells were observed daily under a microscope for the appearance of PPRV-specific CPE. Rescued viruses were further passaged at least three times before stocks of viruses were prepared for further study.

### 2.8. Virus Characterization

RT-PCR was carried out on the total RNAs isolated from virus-infected Vero/VDS cells in order to characterize the chimeric viruses. The N-gene was amplified using the specific primer sets initially used for the amplification of the chimeric N-genes (see Supplementary Table S3). The PCR products were sequenced on both the strands to confirm they were from the desired virus. Multistep growth curves and plaque morphology assays of the parent and recombinant viruses were carried out, as previously described [10].

### 2.9. Growth of Recombinant Virus in Tissue Culture

In order to determine whether swapping a part of the N-protein gene of PPRV with parts from the DMV had any deleterious effect on virus replication, multi-step growth curves were carried out by infecting VDS cells in 6-well plates at approximately 70% confluency, with equal MOIs of the recombinant and parental viruses. Viruses were allowed to adsorb the cell monolayers for 1–2 h and the unbound virus was removed by washing the cells three times with 2 mL of growth medium. Finally, 2 mL of growth medium was added to each well and the cells were incubated for different time periods (0, 12, 24, 36, 48, 60, 72, 84, and 96 h post-infection). Each virus growth curve was carried out in duplicate and, at each time point, the infected cells were frozen at –70 °C. The virus was harvested after one cycle of freeze-thawing and the titer of the released virus was determined by measuring TCID50 on Vero/VDS cells. The log_10_ of the titer obtained was plotted against each time point.

### 2.10. Confocal Fluorescence Microscopy

In order to study the distribution pattern of the viral proteins in infected cells, confocal microscopy was carried out, as described earlier [10]. Briefly, Vero cells, on coverslips, were infected with recombinant viruses and fixed at approximately 48 h post-infection and permeabilized. The non-specific binding of antibodies to cells was blocked by incubating the cells for 5 min in Ca^2+^/Mg^2+^ free PBS containing 0.2% of gelatin. The cells were then labeled for surface or internal proteins using mouse monoclonal antibodies against the PPRV protein as required. The primary antibodies were detected using Alexa Fluor 488 conjugated goat anti-mouse IgG and Alexa Fluor 546 conjugated goat anti-rabbit IgG (molecular probes). The coverslips were mounted using Vectashield (Vector Laboratories, Burlingame, CA, USA) and the cells were imaged on a confocal microscope-Leica TCS SP2 (Leica, Wetzlar, Germany) using sequential scanning. The resultant TIFF files were resized and color overlays prepared with Adobe Photoshop.

### 2.11. Animal Studies

#### 2.11.1. Ethics Statement

Two separate animal experiments were carried out during this study in the high containment facilities at the Pirbright Institute (TPI), Pirbright, United Kingdom. The animal experiments were approved by the Animal Welfare and Ethical Review Board (AWERB) of TPI and conducted according to the guidelines of the UK Home Office project license number 70/6907, approved on 18/12/2015.

#### 2.11.2. In Vivo Vaccination and Challenge Experiment

The recombinant viruses (rPPRV–DIVA: rSungri/96-DMV and rNigeria/75/1-DMV) and the parent vaccine viruses (PPRV/Sungri/96 and PPRV/Nigeria/75/1) were used for vaccination purposes, whereas the virulent viruses PPRV/Ghana/78/1 and PPRV/Morocco/2008 [24] were used as the challenge viruses in this study. For each vaccine strain, 15 goats of either sex, aged from 6 to 12 months, were randomly divided into 3 groups (*n* = 5/group). The animals were kept under observation for at least a week, for acclimatization purposes, in the isolation unit at the TPI prior to vaccination. The animals in groups 1 and 2 were vaccinated subcutaneously with 10^5^ TCID_50_ of rPPRV–DIVA and PPRV conventional vaccines, respectively. Group 3 served as the unvaccinated controls. At 4 weeks post vaccination, all the animals were challenged with a virulent PPRV; PPRV/Ghana/78/1 was used for the Sungri/96 vaccine experiment (10^5^ TCID_50_), whereas PPRV/Morocco/2008 was used for the Nigeria/75/1 vaccine experiment (10^5^ TCID_50_) via the intranasal route (volume of 1 ml/nostril) using an LMA^®^ MAD Nasal™ Intranasal Mucosal Atomization Device (LMA, San Diego, CA, USA) to mimic natural infection. The recording of rectal temperatures and clinical assessments of animals were conducted twice daily. Following challenge, the animals were treated with antibiotics on day 5 to avoid the occurrence of a secondary infection. The clinical scores for each animal were calculated, taking into account the rectal temperature and other clinical signs [25], and the animals that developed severe clinical signs were humanely killed.

Clinical samples (heparinized and clotted blood samples, and ocular, nasal, and saliva swabs) were collected on days 0, 7, 14, 21, and 28, following vaccination, and taken daily or on alternate days, following challenge. Heparinized blood samples were used for the leukocytes count, whereas clotted blood was used for the separation of serum that was subsequently used for the detection of anti-PPRV antibodies. The serum samples were stored at −20 °C until use, whereas the swab samples were stored at −70 °C until processed.

### 2.12. Assays for Anti-PPRV Antibodies

The development of PPRV neutralizing antibodies was measured using a virus neutralization test (VNT), as previously described [12]. The titer was calculated as the reciprocal of the dilution at which 50% of wells showed no CPE and was expressed as log_10_ TCID_50_/mL. The titers of anti-N antibodies in the serum were determined using a commercially available competitive ELISA kit (IDVet, Grabels, France), as described by the manufacturer.

### 2.13. Detection of PPRV RNA in Clinical Samples

The total RNA was isolated from nasal, ocular, and oral swabs, as described previously [24], using the KingFisher Flex automated extraction platform (Thermo Fisher Scientific, UK) with the MagMAX-96™ viral RNA isolation kit (Thermo Fisher Scientific, UK), and the RNA eluted in a final volume of 90 µL. All RNA samples were stored at −70 °C as single use aliquots until tested. All ocular, nasal, and mouth swabs, and blood and fecal samples, were analyzed by real-time RT-PCR (RT-qPCR) to assess the viral load [23] using the Superscript III Platinum R one step qRT-PCR system kit (Invitrogen, Carlsbad, CA, USA) on the ABI 7500 system (Applied Biosystems, Paisely, UK). All samples were run in duplicate and the samples showing positive results in only one well were repeat tested for confirmation.

### 2.14. Development of an Indirect ELISA to Detect PPR-DIVA N-Specific Antibodies Using the Recombinant N-Protein

An indirect ELISA was developed using *E. coli* expressed recombinant proteins (PPRVNv or DMVNv) as the antigen. Plates were coated with the protein (1 mg/mL) at a 1:2000 dilution in a carbonate/bicarbonate buffer (pH 9.6) for 1 h at 37 °C. After washing it with PBS, the serum sample was added at a 1:8 dilution in a blocking buffer containing 0.1% of Tween-20 and 5% of Marvel in PBS and incubated for another hour. After a thorough wash, horseradish peroxidase-conjugated anti-bovine IgG (Sigma) was added at a dilution of 1:5000 in a blocking buffer. After an hour of incubation at 37 °C, the plates were washed and the substrate (OPD) solution was added for the development of color. Color development was stopped after 10 min by adding 1M of sulfuric acid. The plate was read in an ELISA plate reader using a 492 mm filter. In order to avoid the background color in the wells of indirect ELISA plates, the net sample optical density (OD) was obtained after deducting the value of a 0-day post-vaccination (dpv) sample from the same animal.

### 2.15. Statistical Analysis

The statistical analysis of the data was carried out using Minitab 18 (Minitab, LCC, State College, PA, USA), and graphs were made using GraphPad Prism version 8.0.1 (GraphPad Software, La Jolla, CA, USA).

## 3. Result

### 3.1. Rescue and Characterization of Recombinant PPRVs from cDNA Clones

VDS cells (~70% confluent) were transfected with the full-length genome plasmids (pSungri/96-GFP; pSungri/96; pSungri/96-DMV_120_; pNigeria/75/1; and pNigeria/75/1-DMV_120_), along with helper plasmids, pN, pP, and pL. Infectious viruses were recovered from all the plasmids. The CPE characteristics of the PPRV infection were observed from four days post transfection (data not shown). The GFP expression in pSungri/96-GFP transfected cells appeared at 3 days post-transfection (data not shown). The CPE observed for all the recombinant viruses appeared as identical to that produced by the parent Sungri/96 or Nigeria/75/1 vaccine viruses. The identities of these rescued viruses were confirmed by RT-PCR, followed by sequencing. The total RNA isolated from the recovered chimeric viruses (rPPRV/Sungri/96-DMV, rPPRV/Nigeria75/1-DMV; henceforth collectively called rPPRV-DIVA) at passage three was subjected to RT-PCR using virus specific primers (PPR-Acl IF/DMV-Pac IR) sets. Parallel reactions were carried out in the absence of the RT enzyme to check for potential plasmid DNA contamination. Amplicons of the expected size (~2 Kb) were obtained with the RT, but no amplified product was observed without RT (data not shown) indicating that the amplification was from the viral RNA. The amplicons were sequenced on both the strands, and the sequences were found to be identical to the respective plasmid sequence.

**Figure 2:**
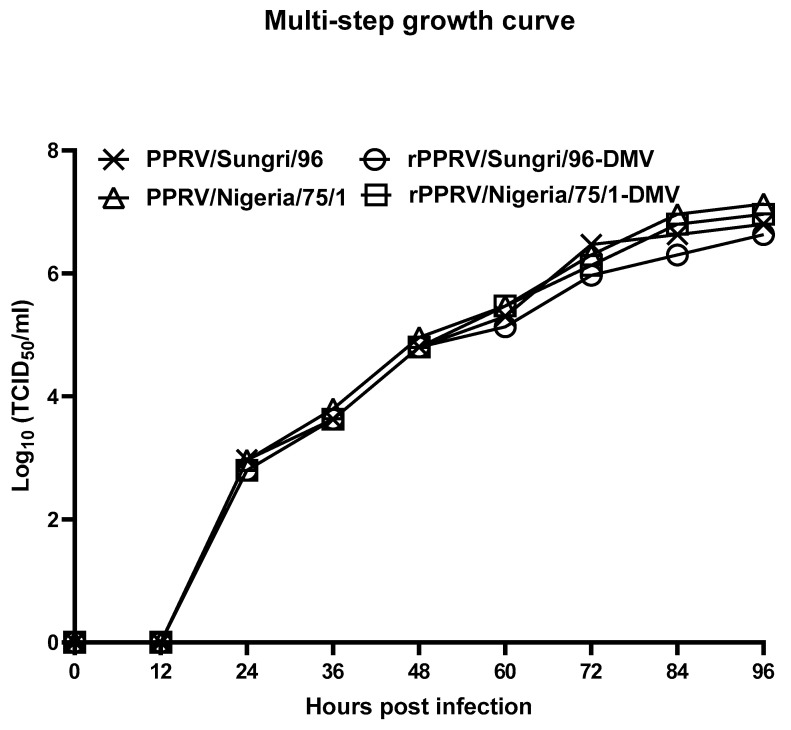
Growth of the recombinant DIVA viruses in cell culture. Growth rates of the recombinant viruses were determined under multi-step growth conditions (MOI = 0.01) for the parental PPRV and two DIVA viruses in the VDS cells.

Multi-step growth curves were carried out to compare the properties of the recombinant viruses (rPPRV/Sungri/96, rPPRV/Sungri/96-DMV, and rPPRV/Nigeria/75/1-DMV) with those of the parent vaccine strains (PPRV/Sungri/96 and PPRV/Nigeria/75/1). The growth characteristics of the rPPRV/Sungri/96 were found to be similar to the parental PPRV/Sungri/96 virus (data not shown). The rPPRV-DIVA viruses grew at a similar rate, and also to a similar titer as that of the parental viruses (Figure 2), indicating that swapping the variable part of the N-gene with that from the DMV did not have any deleterious effects on the replication efficiency of the recombinant viruses.

### 3.2. Confocal Microscopic Studies

**Figure 3:**
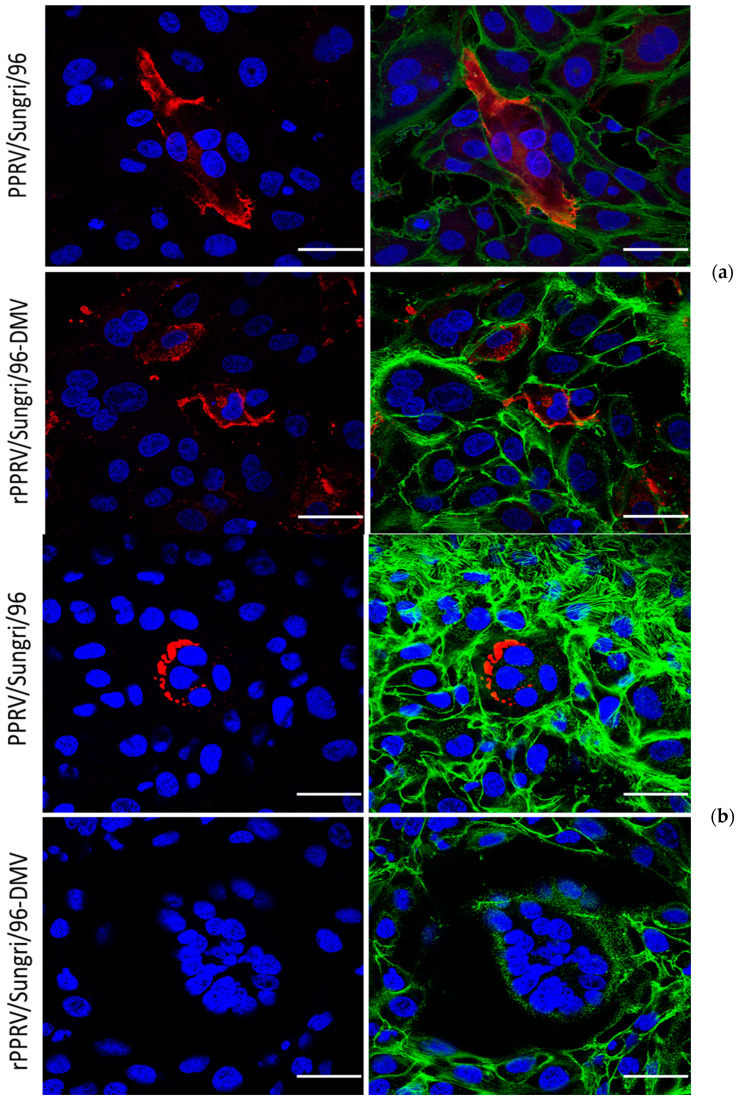
(**a**) Expression of the H-protein in cells infected with the parental PPRV/Sungri/96 and rescued rPPRV/Sungri/96-DMV viruses. A similar pattern was observed in PPRV/Nigeria/75/1 and rescued rPPRV/Nigeria/75/1-DMV virus infected cells. (**b**) Expression of the N-protein in cells infected with the parental PPRV/Sungri/96 and rescued rPPRV/Sungri/96-DMV viruses. A similar pattern was observed in PPRV/Nigeria/75/1 and rescued rPPRV/Nigeria/75/1-DMV virus infected cells. The scale bar represents 25 µm.

Two monoclonal antibodies, one against the PPRV H-protein (C-77) (as used by [12]) and one against the PPRV N-protein (N-C1), were used in this study. The N-C1 mAb was raised against the variable C-terminal region of the PPRV N-protein. VDS cells infected with either PPRV/Nigeria/75/1, PPRV/Sungri/96, rPPRV/Nigeria/75/1-DMV, or rPPRV/Sungri/96-DMV were stained with the primary antibody followed by Alexa Fluor^TM^ conjugated anti-Mouse IgG-568. To observe the cell boundary, the cells were simultaneously stained with an actin stain, Alexa Fluor^TM^-488 phalloidin. The confocal microscopic results demonstrated the normal distribution of the PPRV H- and N-proteins in the cells infected with parent viruses (Figure 3a,b). In contrast, no N-protein staining was observed in the rPPRV/Sungri/96-DMV virus infected cells, although the H-protein distribution was found to be normal (Figure 3a,b). This indicates that mAb N-C1 specifically detects only the PPRV N-protein and does not recognize the DMV N-protein (Figure 3b). Since PPRV specific syncytia were observed in cells infected with both parent and recombinant viruses, and both were stained with anti-H monoclonal C-77, the absence of staining by mAb N-C1 in the rPPRV/Sungri/96-DMV virus confirmed that the C-terminal variable region of the N-protein was not from PPRV in the recombinant viruses. Similar results were obtained when cells infected with the PPRV/Nigeria/75/1 and rPPRV/Nigeria/75/1-DMV viruses were stained with anti-H and anti-N mAbs (data not shown).

### 3.3. In Vivo Evaluation of rPPRV-DIVA Vaccines

**Figure 4:**
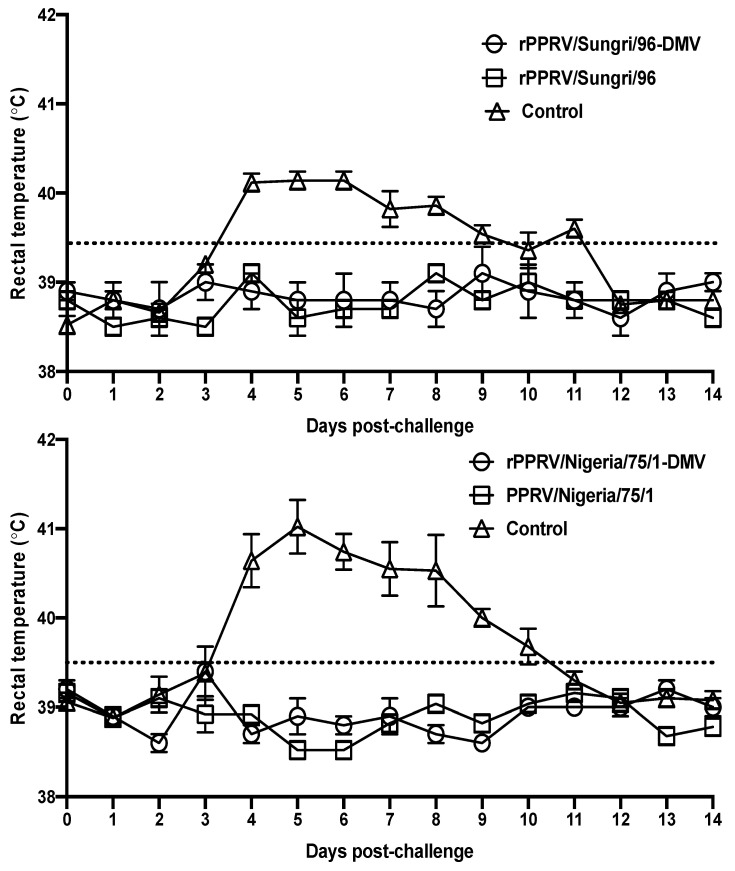
Rectal temperatures of vaccinated (recombinant and parent vaccine virus) and unvaccinated goats upon challenge with the virulent PPRV. The temperatures were measured twice per day and are presented as the mean values of the five animals in each group with a standard error. After 10 days post-challenge (dpc), the control group data are presented as the mean values of 2 and 4 animals in the Sungri/96 and Nigeria/75/1 experimental groups, respectively.

To study the effectiveness of the chimeric viruses as vaccines, preliminary vaccination trials were carried out in the natural host (goat) by comparing it with the parent PPRV/Sungri/96 and PPRV/Nigeria/75/1 vaccines. No vaccination-associated side effects were observed in any of the animals vaccinated with rPPR-DIVA or conventional vaccines at any time during this study. The animals in both the vaccinated groups remained healthy during the post-challenge period, whereas all five animals in the control group developed PPR disease-specific clinical symptoms (pyrexia, congested oro-nasal mucosa, mucopurulent nasal discharge, conjunctivitis, diarrhea, and anorexia). Following the vaccination, there was no rise in the rectal temperatures in any of the animals; following challenge the result was the same for all the vaccinated animals, whereas a rise in rectal temperature was observed in the control animals in both experiments (Figure 4). The onset of disease in control animals was noticed from 4 dpc, and a significant rise in rectal body temperatures (>40 °C) was observed until 8 dpc in some animals, with the peak temperatures observed on days 4 and 5 post-challenge. All the vaccinated animals maintained their rectal temperatures within the normal range throughout the study period and showed no clinical symptoms. Due to severe clinical disease, 3 animals in the control group in the Sungri/96 experiment and 1 animal in the control group in the Nigeria/75/1 experiment had to be humanely killed on 10 days, post-challenge.

**Figure 5:**
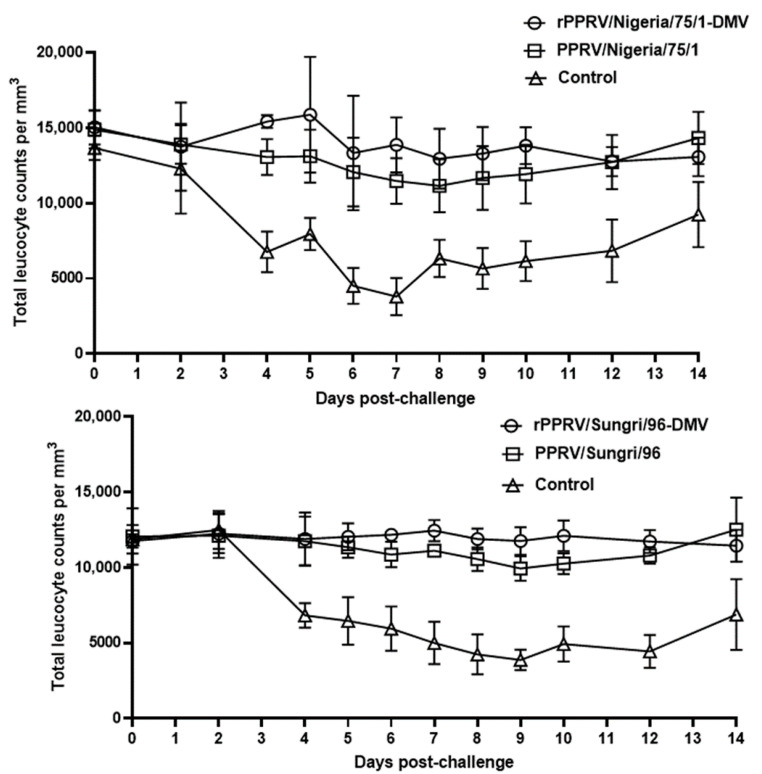
Leukocyte counts for the vaccinated and unvaccinated goats on the virulent PPRV challenge. The total number of leukocytes were counted using a hemocytometer and expressed as the total number of WBCs per ml of blood. The mean values of five animals in each group with ± standard error of mean is shown. After 10 days post-challenge, the control group data are presented as the mean values of 2 and 4 animals in the Sungri/96 and Nigeria/75/1 experimental groups, respectively.

Vaccination with the rPPRV-DIVA and conventional live attenuated vaccines did not show any immunosuppressive effects in the animals exposed to vaccine viruses and virulent PPRV (Figure 5), as there was no evidence of decreased leukocyte counts. However, a severe decrease in the leukocyte count was observed in the animals in the control group from day 4 post-challenge (Figure 5).

### 3.4. Viral RNA Detection in the Clinical Samples

Clinical samples, collected from swabs taken from the nose, mouth, and eyes, following challenge were analyzed by RT-qPCR to determine the level of virus replication and excretion in vaccinated and unvaccinated goats. No viral nucleic acid was detected in the vaccinated groups (rPPR-DIVA and PPRV) throughout the challenge period, whereas viral nucleic acid was detected in the unvaccinated control animals from day 4–12, post-challenge (data not shown).

### 3.5. Neutralizing Antibody Titers

**Figure 6:**
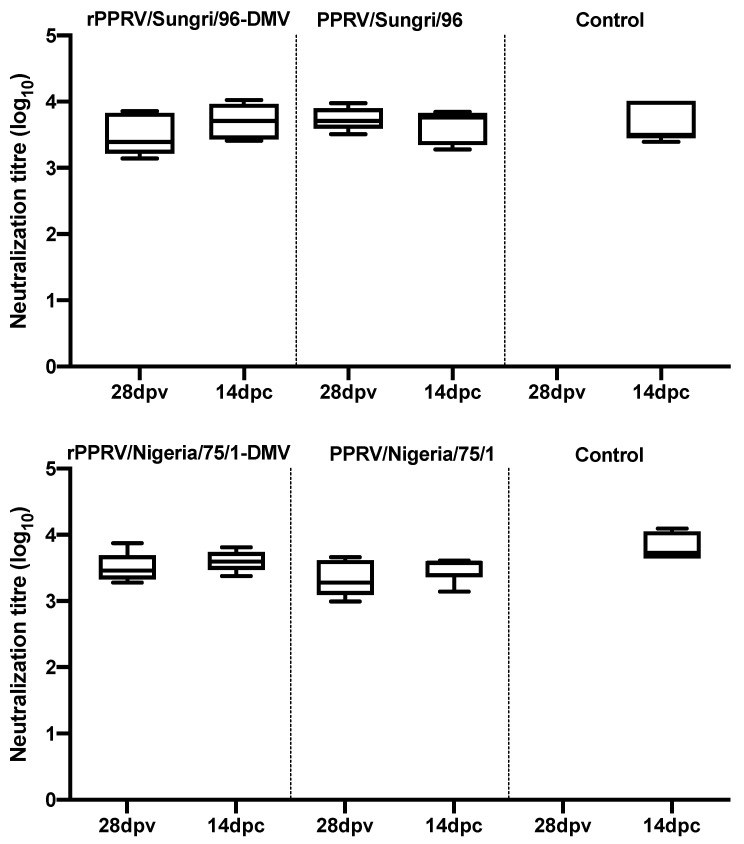
The antibody response in goats to PPRV vaccines; dpv: days post-vaccination; dpc: days post-challenge The neutralizing titers of the sera collected on 28 dpv and 14 dpc were determined against the Nigeria/75/1 and Sungri/96 vaccine viruses. The data are presented as box-and-whisker plots, in which the bars span the minimum and maximum values, and the box shows the range from the first to the third quartile. The central horizontal line in each box shows the median value. The 14 dpc control group data are from 2 and 4 animals in the Sungri/96 and Nigeria/75/1 experimental groups, respectively.

Serum neutralization assays were carried out to determine the virus neutralizing antibody titers, if any, in the serum of vaccinated and challenged animals. High titers of virus neutralizing antibodies were observed in both the vaccinated groups. No significant differences between the antibody titers were observed in the groups vaccinated with conventional or DIVA vaccines on the day of challenge (Figure 6). Following challenge, there were no further increases in the neutralizing antibody titers, indicating that the vaccines provided sterile immunity.

### 3.6. Detection of N-Specific Antibodies Using Existing cELISA

**Figure 7:**
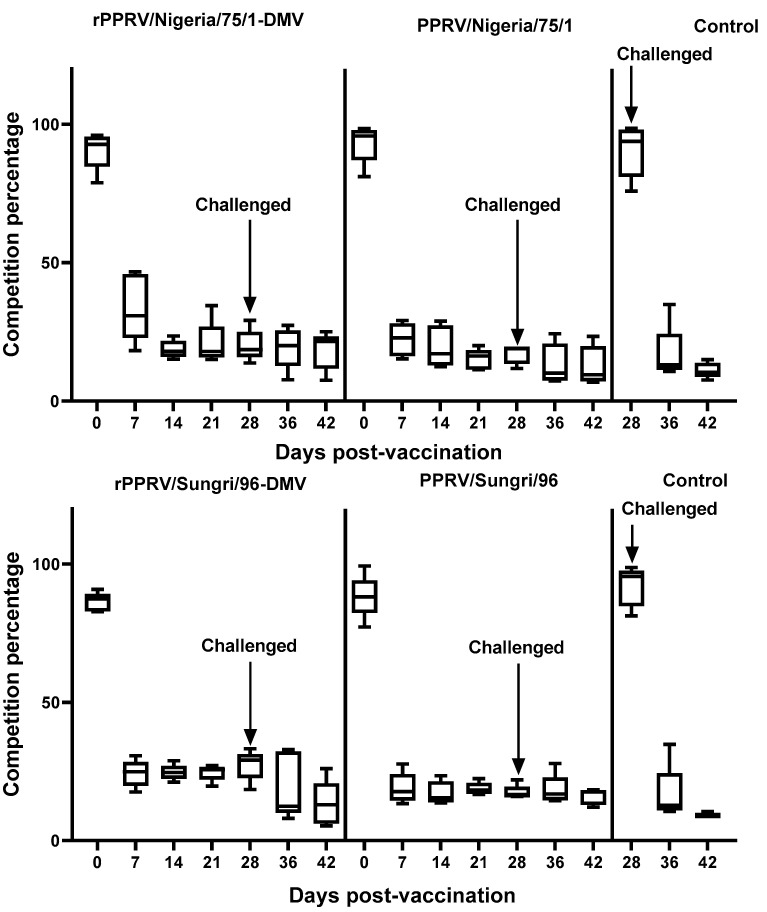
Antibody response in goats to PPRV vaccines. Goats were vaccinated with the recombinant and parental vaccine strains and samples were taken at 0, 7, 14, 21, 28, 36 and 42 days post-vaccination (dpv). Sera taken at all the time points were assayed for antibodies to the PPRV N-protein using the commercially available cELISA kits. Note that the manufacturer’s recommended calculation for the N-protein cELISA kit gives values that decrease as the amount of competing antibodies increases (percent inhibition of binding of mAb). The data are presented as box-and-whisker plots, in which the bars span the minimum and maximum values, and the box shows the range from the first to the third quartile. The central horizontal line in each box shows the median value. The 42 dpv control group data are from two and four animals in Sungri/96 and Nigeria/75/1 experimental groups, respectively.

cELISA was carried out on sera collected from the experimental animals to measure the PPRV N-specific antibody response, using the commercially available N-protein-based cELISA kit as described in the Methods section. All animals vaccinated with either PPRV or rPPRV-DIVA exhibited less than 50% inhibition of N-specific antibodies from 7 days post-vaccination (Figure 7). Following challenge, comparable levels of N-protein specific antibodies were detected in both groups of animals that received either rPPRV-DIVA or conventional PPRV vaccines, suggesting that the existing N-protein-based cELISA did not differentiate between the vaccinated and infected animals.

### 3.7. Cloning and Expression of the C-Terminal Variable Region of PPRV and DMV N-Protein

**Figure 8:**
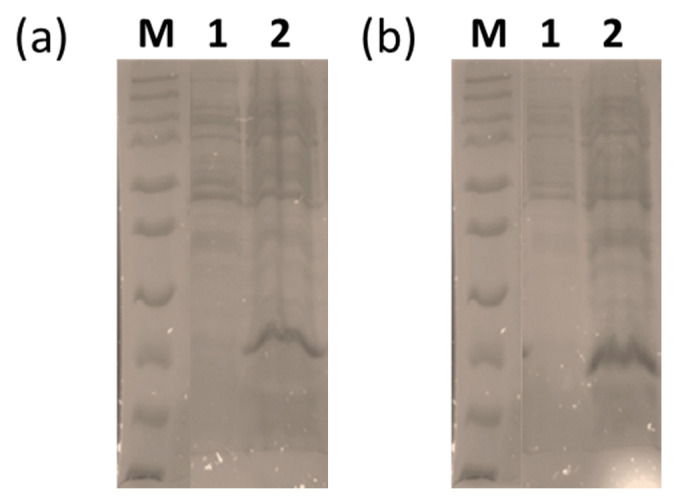
Uninduced and induced cell lysates from *E*. *coli* were run on 12% SDS PAGE. (**a**) M-protein marker, lane 1 uninduced PPRVN protein; lane 2 induced PPRVN protein; and (**b**) M-protein marker, lane 1 uninduced DMVN protein; lane 2 induced DMVN protein.

In order to develop a more virus-specific ELISA, the coding regions for the C-terminal variable region of PPRV and DMV N-proteins (PPRVNv and DMVNv) were cloned into the bacterial expression vector pQE30Xa. The N-terminal His-tagged proteins were expressed efficiently from this vector in *E. coli* (Figure 8) and found to be soluble in nature. Both the recombinant proteins were purified using Ni-NTA resin to near homogeneity (data not shown).

### 3.8. Serological Differentiation of Animals Vaccinated with the rPPR-DIVA Vaccine from Animals Vaccinated with a Conventional Vaccine

**Figure 9:**
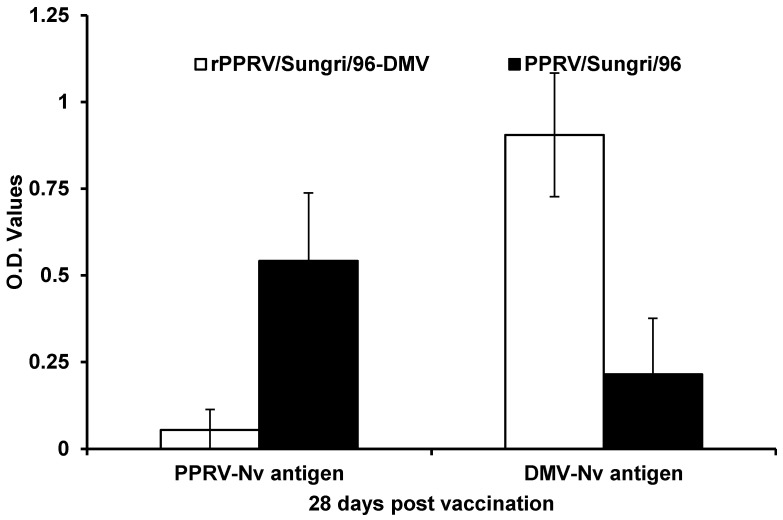

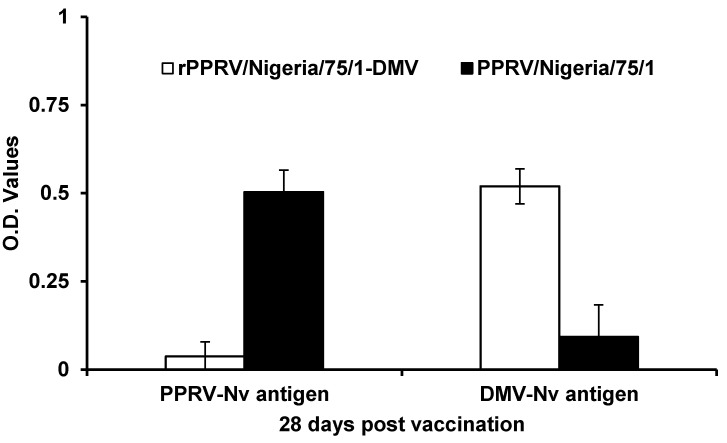
Mean optical density against the native PPRV N-protein and DMV N-protein in DIVA ELISA. The assay was carried out using 28 days post-vaccination serum samples.

The PPRV N-protein specific antibody response in serum collected from the vaccinated animals was assessed using two recombinant proteins: (i) the native PPRV N-protein (PPRVNv) and (ii) the DMV N-protein (DMVNv) in the DIVA ELISAs. The PPR ELISA using the PPRVNv protein as an antigen could detect the animals vaccinated with conventional vaccines and the infected animals in the control groups, whereas the ELISA using the DMVNv antigen only detected animals vaccinated with the rPPRV-DIVA vaccines (Figure 9).

## 4. Discussion

Across the developing world, PPR, usually called the “goat plague”, places a huge burden on agriculture, particularly affecting sheep and goat production. The disease is widely prevalent in Africa, Asia, and the Middle East, where small ruminants play an integral part in sustainable agriculture, and PPR affects the livelihoods of the poorer sections of society. PPR across the endemic areas has been mainly controlled using two conventional live attenuated PPRV vaccines (PPRV/Nigeria/75/1 and PPRV/Sungri/96). The PPRV/Nigeria/75/1 strain is used in endemic countries all over the world, except in India, whereas the PPRV/Sungri/96 vaccine is mainly used in the Indian sub-continent. However, due to the lack of an appropriate vaccination strategy in endemic countries, PPR has emerged as an important viral disease with serious economic consequences for small ruminant production. PPR is currently the focus of a global eradication program; however, no tools currently exist that allow the serological differentiation between vaccinated and infected animals. Marker vaccines are a potential solution, to differentiate between vaccinated and infected animals, which would be immensely helpful in PPR control programs, at least at the last phase of eradication. Without a marker vaccine and associated DIVA tests, the disease can take longer to be eradicated as can be observed from the rinderpest eradication program [1,26].

Using reverse genetics, we previously recovered a recombinant PPRV/Nigeria/75/1 vaccine virus from a cDNA clone [12]. Furthermore, in that study, the epitope of C77 mAb (used in the commercially available H-cELISA kit) was changed in the genome with the aim of developing a DIVA vaccine for PPR. This vaccine was assessed in vivo in goats and found to be potent, safe and efficacious. However, the DIVA strategy was found to be unsuccessful, probably due to the interference of antibodies generated against overlapping epitopes in the polyclonal serum, indicating the need for the development of a DIVA vaccine with the change of a longer fragment of the gene that may solve the issue, as observed in the previous study [12], and can further help in the global PPR eradication program. In the current study, we report the establishment of a reverse genetics system for the lineage IV Indian vaccine strain PPRV/Sungri/96 for the first time and, furthermore, the replacement of the variable part of the N-gene (encoding the last 120 aa) of both the PPRV vaccine viruses (PPRV/Sungri/96 and PPRV/Nigeria 75/1) with the equivalent region from the dolphin morbillivirus, to develop two marker vaccines (Sungri/96-DIVA and Nigeria/75/1-DIVA).

The C-terminal tail region of the paramyxovirus N-protein is variable and has been reported to be dispensable for RNA binding; however, it interacts directly with viral M-proteins [27,28,29,30,31] and P-proteins [32,33,34]. In addition, in paramyxoviruses, the glycoprotein tails and the nucleocapsid interact with each other through the matrix protein. Hence, it was expected that the replacement of the variable region of the N-protein of the PPRV virus with that from the DMV may result in a non-homologous interaction between the N- and the M-proteins, which can affect the growth and phenotypic characteristics of the chimeric viruses as observed in the rRPV-PPRMFH virus [10]. Surprisingly, infectious viruses were recovered from the two full-length genome plasmids, and the replication efficiency and phenotypic characteristics of both the recombinant chimeric viruses were found to be similar to their respective parental viruses, indicating that the replacement of the variable part of the N-gene from a related morbillivirus did not have much impact on the growth, phenotypic characteristics, and N- and H-protein distribution in infected cells. This was similar to the reports in RPV and NDV, in which the entire N-gene or a small epitope was replaced [20,21]. Therefore, this study reveals that the exchange of the C-terminal part of the N-protein of PPRV from a related morbillivirus is tolerated. Furthermore, this fact is supported by earlier reports that presented the exchange of N-proteins between two different morbilliviruses, i.e., rinderpest and PPRV [20]; between a morbillivirus, measles virus (MV), and a pneumovirus, Respiratory Syncytial Virus (RSV) [35]; and, also between two related paramyxoviruses, bovine parainfluenza virus 3 (BPIV3) and human parainfluenza 3 (HPIV3) [36] were possible without any deleterious effect on the growth, replication efficiency, and immunogenicity of the chimeric viruses. Furthermore, deletion/replacement of a conserved B cell immunodominant epitope at the C-terminus of the N-protein (residues 443–460) in the Newcastle Disease Virus (NDV) had no significant impact on the replication efficiency of the virus, indicating that the epitope is not only dispensable for virus replication, but also can be replaced by foreign sequences [21].

The rescued rPPRV-DIVA vaccine viruses were passaged repeatedly and sequenced to confirm that the replacement of the variable part of the N-gene from the DMV was genetically stable, at least for up to seven passages. Liu and colleagues also reported the rSRMV-eGFP to be genetically stable for at least up to five passages [37]. In a recent report, Clarke and colleagues reported a very slow evolution of the PPRV genome [38], which probably explains why these recombinant viruses are genetically stable. A pilot vaccination trial in goats was conducted to evaluate the rPPRV-DIVA viruses as marker vaccines, and to compare their safety and efficacy with the tissue culture-attenuated parent vaccine viruses. Vaccinated animals did not show any clinical signs specific for PPR either during the vaccination or following challenge, similar to previous reports in which animals were vaccinated with either the PPRV/Nigeria/75/1 or PPRV/Sungri/96 vaccines [39]. The control animals were housed with the vaccinated animals, sharing food and water supplies throughout the vaccination period of this study, but did not show any evidence of PPR disease-specific clinical signs or sero-conversion on or before the day of challenge, indicating that there was no transmission of the vaccine virus during the vaccination period. Although there are not many published reports on the transmission of the PPRV/Sungri/96 vaccine, the results of a previous study [40] demonstrate that the PPRV/Sungri/96 vaccine is highly unlikely to transmit between animals. Similarly, studies by Muniraju and colleagues, involving the testing of recombinant PPRV/Nigeria/75/1 (rPPRV-C77) in goats, revealed that the recombinant modified version of the virus did not produce any PPRV-specific clinical signs in the vaccinated animals, and upon sequencing of the virus recovered from the PBMCs of vaccinated animals no mutations were observed [12]. In this study, no unwanted mutations were detected after passaging these viruses seven times in the cell culture. Therefore, it is clear that the vaccines used in this study were safe to use.

Following an intranasal challenge with a highly virulent PPRV on 28-day post-vaccination, all the vaccinated goats were fully protected; in contrast, all the unvaccinated control animals developed pyrexia within 48 h of challenge and, subsequently, developed severe clinical diseases. The vaccines (both DIVA and parent) used in this study provided sterile immunity, as previously observed [40,41]. Neutralizing antibodies were detected in all the vaccinated animals, and animals vaccinated with rPPRV-DIVA vaccines exhibited comparable neutralizing antibody titers to those induced by the parent tissue culture adapted PPR vaccines. Muniraju and colleagues also reported high titer PPRV-specific serological responses in PPRV/Nigeria/75/1 vaccinated animals on 28-days post-vaccination, by both VNT and ELISA [12]. In this study, rPPRV-DIVA vaccinated animals generated a considerable antibody response to the homologous virus by 7 days post-vaccination, whereas Hodgson and colleagues could detect neutralizing antibody responses against the PPRV/Sungri/96 virus only from 14 days post-vaccination [40]. This might be due to the lower vaccine dose (2 × 10^4^ TCID_50_) used in the latter study, compared to higher dose (10^5^ TCID_50_) used in our study.

The results of the commercially available N-cELISA showed that all of the vaccinated animals tested strongly positive for PPRV N-specific antibodies. However, this N-protein-based cELISA did not distinguish between animals vaccinated with conventional vaccines, infected unvaccinated animals, and rPPRV-DIVA vaccines, and therefore was not suitable for use as a serological test that could differentiate between vaccination and infection. To circumvent this problem, two recombinant proteins, PPRVNv and DMVNv, were used to develop two indirect ELISAs. The newly developed PPRVNv ELISA did not detect antibodies induced by the rPPRV-DIVA, while the DMVNv ELISA could not detect antibodies induced by the parent PPRV vaccines and infected unvaccinated control animals. Therefore, these indirect ELISAs are suitable for use as companion diagnostic tests in conjunction with these recombinant chimeric vaccines. As the rPPRV-DIVA vaccine lacks the highly antigenic C-terminal part of the PPRV N-protein, it can be used as a marker vaccine to distinguish, serologically, between vaccinated and naturally recovered animals using these two newly developed ELISAs. Animals vaccinated with the DIVA vaccines will be positive in the DMVNv ELISA and negative in the PPRVNv ELISA, whereas animals vaccinated with conventional tissue culture vaccine or infected with the wild-type virus will test positive only in the PPRVNv ELISA and negative in the DMVNv ELISA (Table 1). The commercially available IDVet N-gene-based cELISA will detect all the vaccinated (conventional and DIVA vaccinated animals) and infected animals as the epitope of the mAb used in this ELISA is located outside of the variable part of the N-protein. Parida and colleagues rescued a chimeric rinderpest virus (rRPV-PPRN) by swapping the N-protein ORF from PPRV and developed an indirect ELISA that could differentiate between RPV- and PPRV-infected animals [20]. However, the two indirect ELISAs developed in this study need to be replaced by mAb-based ELISAs in order to improve the assay sensitivity and specificity.

In conclusion, the chimeric rPPRV-DIVA vaccines were found to have DIVA properties, and to be safe and efficacious in goats with similar potency as the parent vaccines. However, large-scale trials involving a much larger number of animals (both goats and sheep) of a different age, sex, breed, and physiological status must be carried out to further establish the safety and potency of these chimeric vaccine viruses, before these can be used in the field. There is currently a plan to take this research forward through PPR vaccine manufacturers and to test these DIVA vaccines in a more significant number of the target animals in the field before commercialization. Similar to a previous report [41], the groups of goats in this study were vaccinated subcutaneously with the Nigeria/75/1 or Nigeria/75/1 DIVA (lineage II) and Sungri/96 or Sungri/96 DIVA (lineage IV) vaccines, and subsequently challenged intranasally with the lineage IV (Morocco/2008) and lineage II (Ghana/78) virulent viruses, respectively, with all animals being protected without any excretion of the virus in any of the body secretions (nasal, eye, and mouth swabs). Therefore, it is clear that both the vaccines (Sungri/96-DIVA and Nigeria/75/1-DIVA) developed in this study can be used in the ongoing global eradication program (GEP), irrespective of the circulation of any lineage (I, II, III, and IV) of the PPR virus.

## Figures and Tables

**Table 1:** Reactivity of the serum collected from vaccinated/infected animals in different ELISAs. +: positive; −: negative.

Serum/ELISA	Commercially Available N-cELISA	PPRVNv ELISA	DMVNv ELISA
Conventional vaccinated/infected animals	+	+	−
DIVA vaccinated animals	+	−	+

## Data Availability

Not applicable.

## Supplementary Materials

The following materials are available online at https://www.mdpi.com/article/10.3390/v13112320/s1, Table S1. Primers designed for sequencing of DMV N-gene; Table S2. Primers designed for overlapping PCR; Table S3. PPRV genome specific primers to RT-PCR; Table S4. Primers designed for expression work.
