# Supplementary material for: Exchange of C-Terminal Variable Sequences within Morbillivirus Nucleocapsid Protein Are Tolerated: Development and Evaluation of Two Marker (DIVA) Vaccines (Sungri/96 DIVA, Nigeria/75/1 DIVA) against PPR
Source: Viruses. 2021 Nov 21;13(11):2320. doi: 10.3390/v13112320 (PMC8623000; doi:10.3390/v13112320)
Supplement: Supplementary file 1 [file viruses-13-02320-s001.zip › viruses-1423437-supplementary.pdf]

viruses

MDPI

Supplementary Materials

Exchange of C-terminal variable sequences within morbillivirus nucleocapsid protein are tolerated: Development and evaluation of a marker (DIVA) vaccine for PPR

Table S1. Primers designed for sequencing of DMV N-gene.

Name of the Primer	Primer Details	Position of Primer
**DMV NF**	GCG CGA TCC TAT CAA TTG GCA CAG G	65–85
**DMV PR**	CCG CTT GAC CGC CGT GAT CAT AAA CAT G	2132<-2157
**DMVNF1**	GAC AGG CGT CAT GAT CAG T	314-332
**DMVNF2**	CCG AGA CGA GGC GGT GGA TTA AA	679-701
**DMVNF3**	GCG AGA CTG CAC CGT ACA TGG TAA	1027-1050
**DMVNF4**	GCT GAG GCA TTA GCC AAG ATG AG	1577-1599
**DMVNR1**	CGG GCG GAT TTT CTC TGA G	1857<-1875
**DMVNR2**	GGT CGT CAG TGT TGT CGG ACT	1535<-1555
**DMVNR3**	CCA ACT CCC ATT GCA TAG CTC CA	1104<-1126
**DMVNR4**	CAC GGC GTT GCT GAG TAT ATT	700<-720
**DMVNR5**	GCC TCT GGA TGC AAA AGT GAG CCC T	443<-467

Table S2. Primers designed for overlapping PCR.

Primer Name	Primer Details	Position of Primer
**PPR-DMV F**	GGC TGG GGA CGA AAG AGC TAA TAG AGC AAT AGG T	1305-1338
**DMV-PPR R**	ACC TAT TGC TCT ATT AGC TCT TTC GTC CCC AGC C	1305<-1338
**DMV *Pac* IR**	CGG CCT TAA TTA AAC GCT GCT CAG AGT GGA TCC	1604<-1585
**PPR *Acl* IF**	GCG CAA GAT CTA ACG TTA TGG CGA CTC TCC	93-120

Table S3. PPRV genome specific primers to RT-PCR.

Primer Name	Primer Position	Primer Sequence
**PPR-F-P6**	2634-2653	AGGAGTGCAAAGACGATCC
**PPR-R-P8**	3830-3850	TTAGCGCTAAACACACTTCC (reverse complementary-GGAAGTGTGTTTAGCGCTAA)

Table S4. Primers designed for expression work.

Primer Name	Primer Details	Position of the Primer
**DMV-*Bam*-HIF1**	GCG GAT CCG CTA ATA GAG CAA TAG GTC C	1315-1340

**DMV-H*ind*IIIR**	GCG CAA GCT TGC CAA GTA GAT CTT TAT C	1665-1690
